# Stability Evaluation and Pharmacokinetic Profiling of Vepdegestrant in Rodents Using Liquid Chromatography–Tandem Mass Spectrometry

**DOI:** 10.3390/molecules29174048

**Published:** 2024-08-27

**Authors:** Hae-In Choi, Jinyoung Choi, Jin Woo Kim, Yoon Ha Lee, Kwan Hyung Cho, Tae-Sung Koo

**Affiliations:** 1 Graduate School of New Drug Discovery and Development, Chungnam National University, Daejeon 34134, Republic of Korea; chi705@naver.com (H.-I.C.); jinyoung724@naver.com (J.C.); dpslzk333@naver.com (J.W.K.); dldbsgk926@naver.com (Y.H.L.); 2 College of Pharmacy and Inje Institute of Pharmaceutical Sciences and Research, Inje University, Gimhae 50834, Republic of Korea; chokh@inje.ac.kr

**Keywords:** vepdegestrant, ARV-471, PROTAC, LC-MS/MS, validation, pharmacokinetics, stability

## Abstract

Vepdegestrant (formerly ARV-471), a novel proteolysis-targeting chimera (PROTAC), targets estrogen receptor alpha (ERα) for degradation, offering a promising option to treat advanced ER-positive breast cancer. We developed and validated a sensitive and rapid liquid chromatography–tandem mass spectrometry method to quantify vepdegestrant in rodent plasma using bavdegalutamide (formerly ARV-110) as an internal standard. Plasma samples were prepared with protein precipitation using acetonitrile and analyzed using reverse-phase C18 columns and a mobile phase of 10 mM ammonium formate in distilled water and acetonitrile. The method demonstrated linearity from 1 to 1000 ng/mL in mouse and rat plasma, meeting all validation criteria, and successfully applied to in vivo and in vitro studies. Pharmacokinetic analysis revealed low-to-moderate clearance (313.3, 1053 mL/h/kg) and oral bioavailability (17.91, 24.12%) of vepdegestrant in mice and rats, respectively. It was unstable in buffer solutions across pH 2–10 and in phosphate-buffered saline (pH 7.4), likely due to adsorption, but remained stable in mouse and rat plasma at varying temperatures. In liver microsomes, vepdegestrant exhibited moderate stability in rats but was stable in mice, dogs, and humans. These findings enhance the understanding of pharmacokinetic properties of vepdegestrant supporting further development of PROTAC drugs.

## 1. Introduction

Breast cancer is one of the most prevalent and challenging malignancies worldwide, with significant morbidity and mortality rates among women. In 2022, breast cancer accounted for approximately 670,000 deaths globally, with 2.3 million women new cases diagnosed [1]. Specifically, the estrogen receptor (ER) has two subtypes, ERα and ERβ, which play crucial roles in the development of breast cancer. Overexpression or mutation of ERα can lead to abnormal amplification of estrogen signaling, potentially leading to cancer development [2]. Endocrine therapies targeting this pathway, including selective estrogen receptor modulators, selective estrogen receptor degraders, and aromatase inhibitors, are the cornerstones of HR+ breast cancer treatment [3]. However, resistance to these therapies poses significant clinical challenges [4,5].

The recent emergence of proteolysis-targeting chimeras (PROTACs) has opened new avenues for the degradation of disease-causing proteins. PROTACs are novel therapeutic strategies designed to selectively remove proteins of interest and target previously “undruggable” proteins [6]. These heterobifunctional compounds consist of a ligand that binds to the target protein and another ligand that binds to an E3 ubiquitin ligase connected by a linker [6,7,8]. PROTACs utilize the ubiquitin–proteasome system, a cellular protein degradation mechanism, to degrade and remove disease-causing proteins [9]. This innovative approach not only inhibits the function of the target protein but also removes it from the cell, potentially overcoming the resistance mechanisms associated with traditional inhibitors [10,11,12].

Vepdegestrant (formerly known as ARV-471), a novel PROTAC drug co-developed by Arvinas and Pfizer, functions as an ER degrader in patients with breast cancer. Vepdegestrant includes lenalidomide as a cereblon (CRBN) ligand, which binds to E3 ubiquitin ligase, whereas the warhead portion selectively attaches to ERα. Vepdegestrant facilitates the selective degradation of ER, disrupting the ER signaling pathway that drives tumor growth and offering a promising therapeutic strategy for patients with locally advanced or metastatic breast cancer [13]. Preclinical studies have demonstrated the potent antitumor activity of vepdegestrant, with significant reductions in ER levels and tumor growth in various breast cancer models [14,15]. Clinical trials have also reported favorable pharmacokinetic (PK) properties, manageable safety profiles, and good clinical activity, indicating its potential as a novel treatment for ER+/HER2− advanced or metastatic breast cancer [16,17]. In addition, a recent study identified metabolites of vepdegestrant in dog microsomes using high-resolution mass spectrometry [18]. It is currently in a phase 3 trial (NCT05654623) under fast-track designation [19]. 

Despite these advancements in phase 3 clinical trials and the Food and Drug Administration (FDA)’s Fast-Track designation in 2024, there are limited data on analytical methods for quantifying vepdegestrant in biological samples or detailed PK studies. Recently, Niessen et al. developed a liquid chromatography–tandem mass spectrometry (LC-MS/MS) method and conducted a PK study in anesthetized rats [20]. However, this study was not peer-reviewed and the animal experiment was conducted with only one subject, raising concerns regarding its reliability. In addition, detailed information on plasma concentrations and PK parameters was not provided, making it difficult to understand the exact characteristics of the drug. Therefore, this study aimed to establish a sensitive and rapid high-performance LC-MS/MS (HPLC-MS/MS) analytical method for the quantification of vepdegestrant, an ER degrader, in mouse and rat plasma. This method was validated according to the guidelines and acceptance criteria of the European Medicines Agency (EMA) and US FDA [21,22]. Furthermore, the developed analytical method can be used to conduct PK studies in mice and rats to understand the in vivo kinetics of the drug. In addition, the stability of vepdegestrant in biological matrices should be evaluated under various conditions to better understand its PK data. This robust and validated analytical method will support future studies and developments, aiding our understanding of the PK and stability profiles of vepdegestrant.

## 2. Results and Discussion

### 2.1. HPLC-MS/MS Method Development

**Figure 1:**
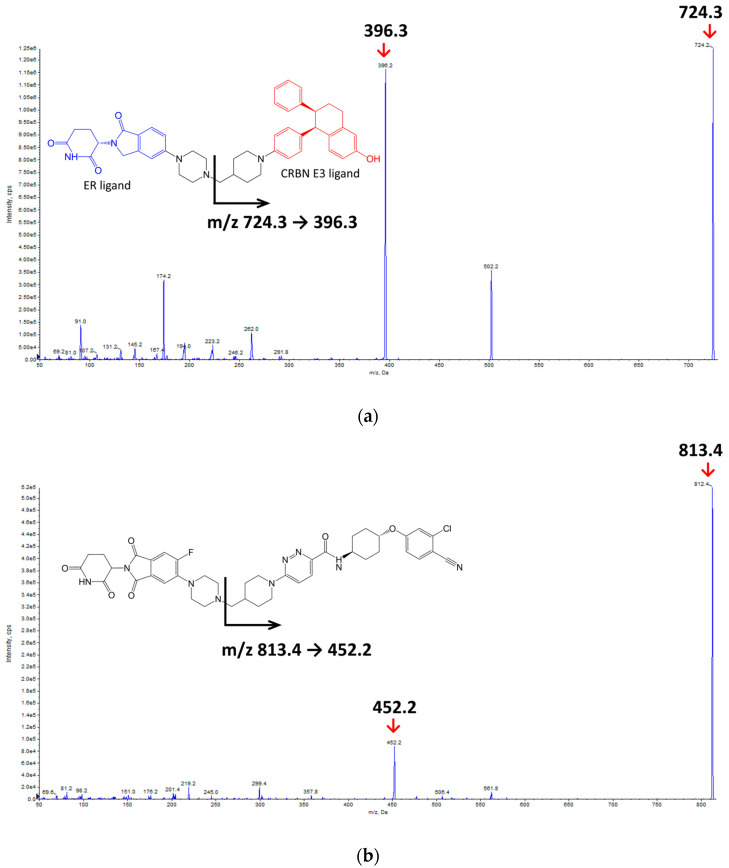
Product ion mass spectra for (**a**) vepdegestrant and (**b**) bavdegalutamide (internal standard; IS).

An Agilent 1100 HPLC series (Agilent Technologies, Santa Clara, CA, USA) and SCIEX API 4000 Qtrap mass spectrometer (SCIEX, Framingham, MA, USA) were used to quantify vepdegestrant in the biological samples. The mass spectra and chemical structures of vepdegestrant (formerly known as ARV-471) and bavdegalutamide (formerly known as ARV-110; internal standard, IS) are shown in Figure 1. The precursor ions were scanned in positive and negative electrospray ionization (ESI) modes to detect the vepdegestrant and the IS. In the Q1 scan, vepdegestrant and IS generated protonated precursor ions [M + H]^+^ with *m*/*z* of 724.3 and 813.4, respectively, in the positive ESI mode, which exhibited superior signal intensity compared to that in the negative mode. Three ion transitions for vepdegestrant were observed: *m*/*z* 724.3 → 174.2, *m*/*z* 724.3 → 396.3, and *m*/*z* 724.3 → 502.2. Among these, the ion transition *m*/*z* 724.3 → 396.3 was the most prominent. For the IS, bavdegalutamide, the transition of 813.4 → 452.2.

The column and mobile phase conditions were optimized to enhance the sensitivity and selectivity of vepdegestrant detection in mouse and rat plasma. C18 and phenyl columns were compared. Vepdegestrant and IS exhibited better peak shapes on the C18 column with no peak tailing, negligible matrix effects, and carry-over. Therefore, the C18 column (Zorbax Eclipse XDB-C18; 3.5 µm, 2.1 × 150 mm, Agilent, Santa Clara, CA, USA) was finally selected. In positive ESI mode, ionization is typically facilitated under weakly acidic conditions. However, owing to inconsistent retention times, broad peak shapes, and severe peak tailing observed when using a mobile phase containing 0.1% formic acid, a combination of 10 mM ammonium formate in distilled water (pH 6.5) and acetonitrile (ACN) was selected as the mobile phase.

### 2.2. HPLC-MS/MS Method Validation

**Figure 2:**
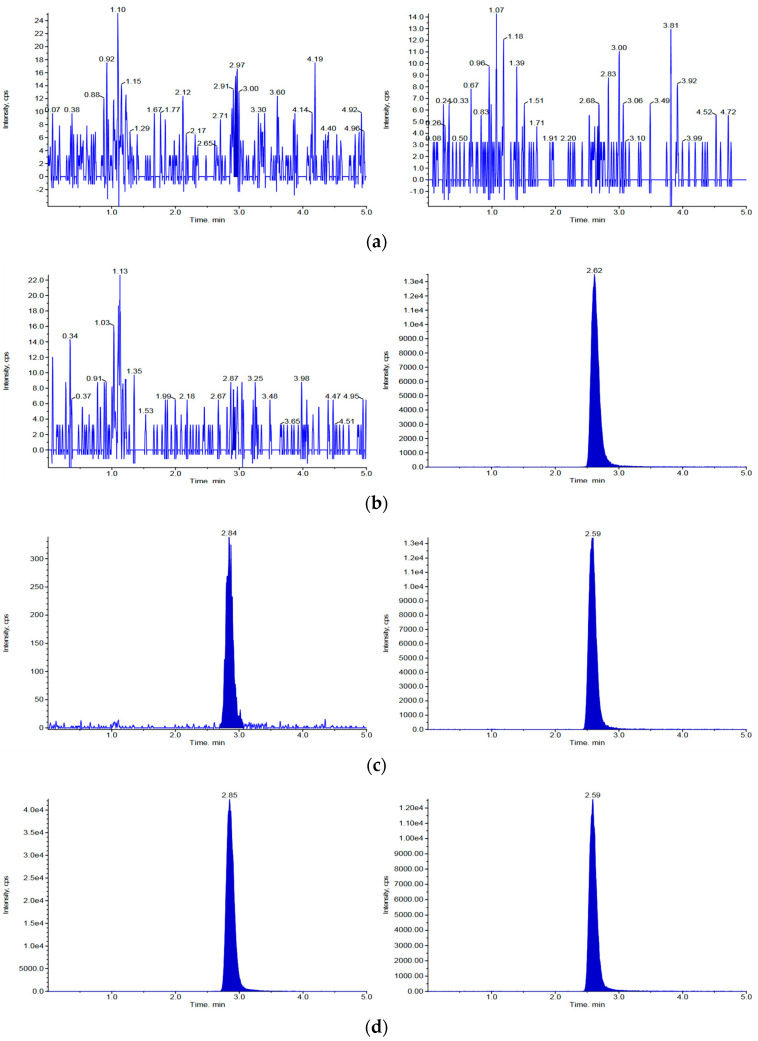
High-performance liquid chromatography-tandem mass spectrometry (HPLC-MS/MS) chromatograms of vepdegestrant and the bavdegalutamide (IS) for mouse plasma. (**a**) Blank mouse plasma absent vepdegestrant and the IS; (**b**) Zero sample, mouse plasma containing only IS (300 ng/mL); (**c**) Mouse plasma spiked with 1 ng/mL (lower limit of quantification; LLOQ) vepdegestrant and IS; (**d**) Incurred sample 15 min following intravenous injection of 2 mg/kg vepdegestrant to mice.

**Figure 3:**
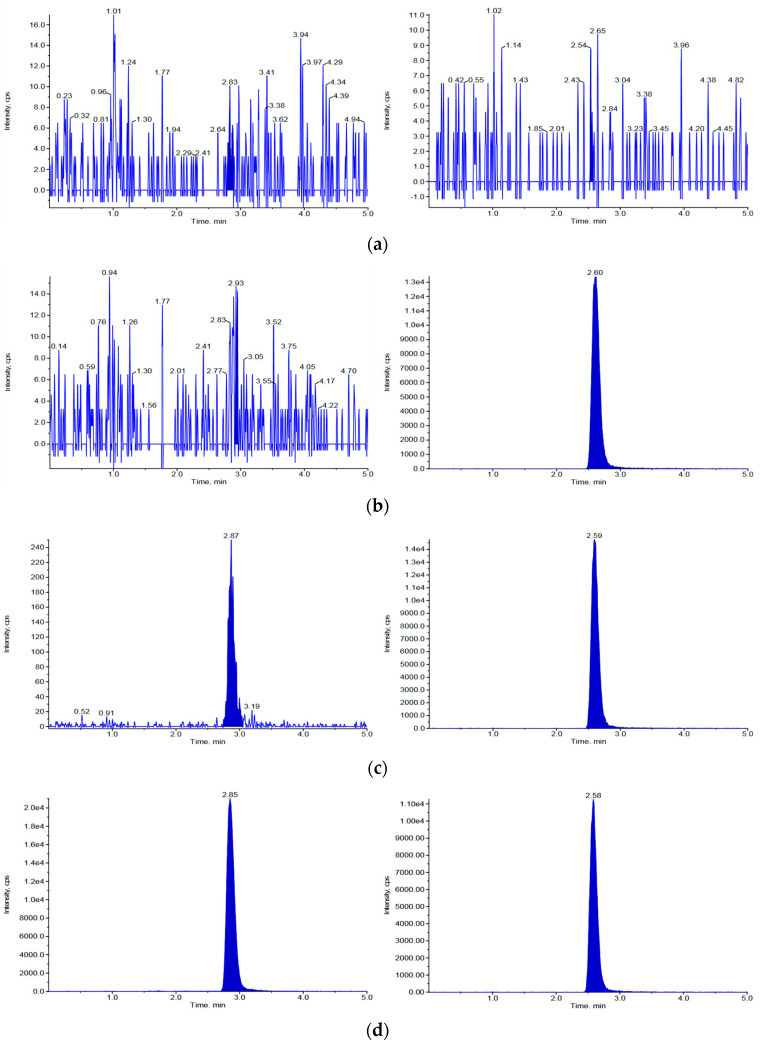
HPLC-MS/MS chromatograms of vepdegestrant and the bavdegalutamide (IS) for rat plasma. (**a**) Blank rat plasma absent vepdegestrant and the IS; (**b**) Zero sample, rat plasma containing only IS (300 ng/mL); (**c**) Rat plasma spiked with 1 ng/mL (LLOQ) vepdegestrant and IS; (**d**) Incurred sample 15 min following intravenous injection of 2 mg/kg vepdegestrant to rats.

The lower limit of quantification (LLOQ) was established at 1 ng/mL in mouse and rat plasma, with signal-to-noise ratios of 9.9 and 8.0, respectively. In mouse and rat plasma, the response at the LLOQ was more than 15.9 and 13.4 times higher than that of the blank plasma, respectively. Chromatograms of blank (no analyte and IS), zero (IS present but no analyte), LLOQ (1 ng/mL), and incurred samples obtained following intravenous injection of 2 mg/kg vepdegestrant in rodents are shown in Figure 2 and Figure 3 for mice and rats, respectively. The retention times of vepdegestrant and bavdegalutamide were 2.84 and 2.58 min in mouse plasma, respectively, and 2.87 and 2.59 min in rat plasma, respectively. As shown in Figure 2 and Figure 3, no interfering peaks were observed at the retention times of vepdegestrant and bavdegalutamide in the blank mouse or rat plasma. Furthermore, consistent retention times were observed in samples from mice and rats. These findings demonstrate the specificity of the analytical method, confirming that vepdegestrant can be detected in mouse and rat plasma, without interference from endogenous substances. 

The calibration curves of vepdegestrant in mouse and rat plasma exhibited good linearity within the 1–1000 ng/mL range using a 1/x^2^ weighting factor. The linear regression equations were y = 0.0246x − 0.000785 (r = 0.9969) for mice and y = 0.028x − 0.00157 (r = 0.9953) for rats.

Table 1 summarizes the intra- and inter-day precision and accuracy results at the LLOQ (1 ng/mL) and three quality control (QC) samples including low QC (LQC; 3 ng/mL), middle QC (MQC; 30 ng/mL), and high QC (HQC; 900 ng/mL). Precision was assessed using the coefficient of variation (CV), and accuracy was evaluated using relative error (RE). In the mouse plasma, the intra- and inter-day precisions were below 10.64% and 13.43%, respectively, whereas the intra- and inter-day accuracies were below 12.00% and 9.04%, respectively. In the rat plasma, the intra- and inter-day precisions were below 10.63% and 13.43%, respectively, whereas the intra- and inter-day accuracies were below 2.10% and 3.84%, respectively. All precision and accuracy results met the acceptance criteria of the FDA and EMA guidelines, demonstrating the repeatability, accuracy, and precision of the HPLC-MS/MS method for vepdegestrant detection in mouse and rat plasma samples.

The stability of vepdegestrant in mouse and rat plasma under various storage conditions is listed in Table 2. The results were within ±15% RE under all storage conditions, including at room temperature for 6 h, after three freeze–thaw cycles, at −20 °C for 4 weeks, and in an autosampler for 24 h after sample preparation. These results indicate that the vepdegestrant is stable under all specified storage conditions.

The matrix effect, recovery, and process efficiency of vepdegestrant and IS in mouse and rat plasma were evaluated using LQC, MQC, and HQC, and the results are summarized in Table 3. In mouse plasma, the values were 87.6–111.3%, 90.7–110.7%, and 91.9–104.9%, respectively (for IS: 92.66%, 94.73%, and 87.78%). In rat plasma, the matrix effect, recovery, and process efficiency were 85.4–107.0%, 84.04–99.26%, and 78.65–94.26% (for IS: 91.99%, 90.05%, and 87.60%, respectively). The matrix effect results suggested that vepdegestrant and IS were not subject to ion suppression from the matrix or mobile phase during the HPLC-MS/MS analysis. Moreover, the high recovery and process efficiencies indicate that protein precipitation is an appropriate pretreatment method for extracting vepdegestrant from mouse and rat plasma.

Dilution integrity was assessed by diluting 10- and 30-fold higher HQC with blank mouse and rat plasma, respectively. For samples diluted 10- and 30-fold with mouse plasma, the CVs were 6.872% and 7.323% and the REs were 8.204% and 10.204%, respectively. For samples diluted 10- and 30-fold with rat plasma, the CVs were 6.166% and 5.658% and the REs were 5.794% and 2.873%, respectively. These results indicate that the dilution effect was negligible in mouse and rat plasma.

### 2.3. In Vitro Stability Tests

**Figure 4:**
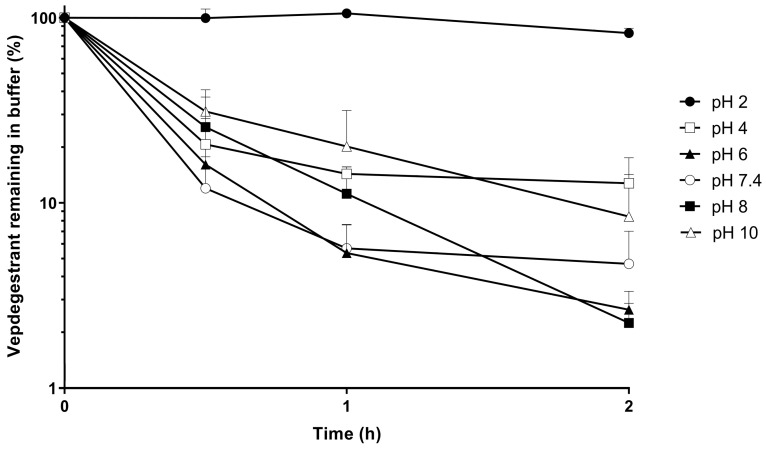
The remaining amount of vepdegestrant (%) in buffer solution (pH 2, ●; pH 4, □; pH 6, ▲; pH 7.4, ○; pH 8, ■; pH 10, △). Data represent the mean ± standard deviation (*n* = 3).

The stability of the vepdegestrant under different pH conditions is shown in Figure 4. Vepdegestrant remained at 82.6% after incubation at 37 °C for 2 h in pH 2 buffer. However, in pH 4–10 buffers, vepdegestrant rapidly decreased within 30 min of incubation at 37 °C, and only 2.25–12.8% of vepdegestrant remained after 2 h, indicating significant instability. The half-lives of vepdegestrant at pH values of 2, 4, 6, 7.4, 8, and 10 were 7.35, 0.77, 0.40, 0.50, 0.38, and 0.60 h, respectively. Sun et al. reported that the thalidomide ligand, a cereblon-targeting moiety of PROTAC, was rapidly degraded in phosphate-buffered saline (PBS; pH 7.4) [23]. The binder in vepdegestrant contained lenalidomide, a CRBN ligand with a chemical structure similar to that of thalidomide. In addition, Chen et al. reported that the glutarimide ring of lenalidomide is cleaved and degraded through non-enzymatic hydrolysis in aqueous solutions and hepatocytes at physiological pH [24]. However, this instability might be attributed to the adsorption of vepdegestrant onto the surfaces of experimental containers such as microtubes. A high proportion of organic solvent can prevent vepdegestrant from binding to the sample vial surface, and the possibility of adsorption exists because a buffer solution was used in this study. To investigate adsorption, after the pH stability test was completed, the incubation mixture was completely emptied, and ACN was added and thoroughly mixed. LC-MS/MS analysis includes MRM transitions for the expected hydrolysis product i.e., m/z 742.3 → 356.3, to detect the presence of degradation products [18]. The recovered vepdegestrant percentages were 21.8%, 108.9%, 105.1%, 72.6%, 63.9%, and 27.7% at pH values of 2, 4, 6, 7.4, 8, and 10, respectively. Slight adsorption was observed at pH values of 2 and 10, whereas extensive adsorption was observed at pH values of 4–8. Additionally, peaks corresponding to hydrolysis products were observed at 1.37 and 1.65 min in all samples, but their exact concentrations could not be determined due to the lack of standards for the hydrolysis products. The +H_2_O peak was significantly larger at pH 10 than at other pH values. These results suggest that the instability of vepdegestrant at pH 2–8 is primarily due to adsorption onto the microtube surfaces rather than degradation. In contrast, hydrolysis may play a significant role at pH 10. Based on these findings, future studies should consider the selection of container materials and the use of organic solvents to minimize vepdegestrant adsorption.

**Figure 5:**
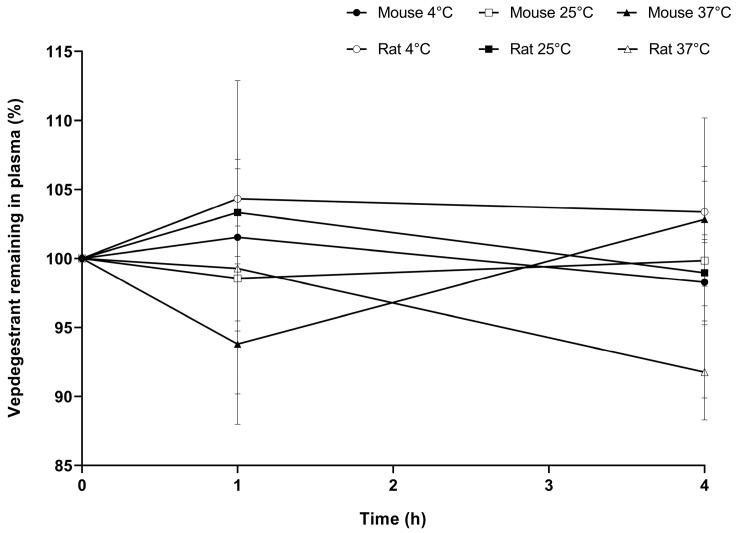
The remaining amount of vepdegestrant (%) in mouse plasma at 4 °C (●), mouse plasma at 25 °C (□), mouse plasma at 37 °C (▲), rat plasma at 4 °C (○), rat plasma at 25 °C (■), and rat plasma at 37 °C (△). Data represent the mean ± standard deviation (*n* = 3).

In contrast, vepdegestrant in mouse and rat plasma did not decrease at 4, 25, and 37 °C for 4 h, with 86.3–113.5% remaining after 4 h of incubation. Figure 5 shows the concentration of vepdegestrant in the plasma as a function of temperature. Low plasma stability was predicted based on pH stability results. However, vepdegestrant exhibited high stability in mouse and rat plasma regardless of temperature changes. These results suggest that strong binding to plasma proteins prevents the adsorption of vepdegestrant onto microtubes. In addition, we evaluated the plasma protein binding (PPB) of vepdegestrant using a rapid equilibrium dialysis device. However, the concentration of vepdegestrant in the buffer was below the quantification limit, making it impossible to determine an accurate binding ratio. This phenomenon persisted even when the administered concentration was increased from 2 to 10 µg/mL or when the plasma was diluted up to 100-fold. Assuming that the buffer concentration was at the lowest quantifiable limit, PPB was calculated to be 99.9%, suggesting that the actual PPB was likely higher. Furthermore, the recovery of vepdegestrant in the plasma after a 4-h incubation was greater than 85%. The unrecovered fraction may result from nonspecific binding to the device and membrane. Further studies should be conducted using alternative methods. Furthermore, unlike bavdegalutamide, which is a CRBN-based PROTAC that is structurally similar, vepdegestrant is expected to exhibit a low probability of degradation by hydrolytic enzymes in the plasma [25].

**Figure 6:**
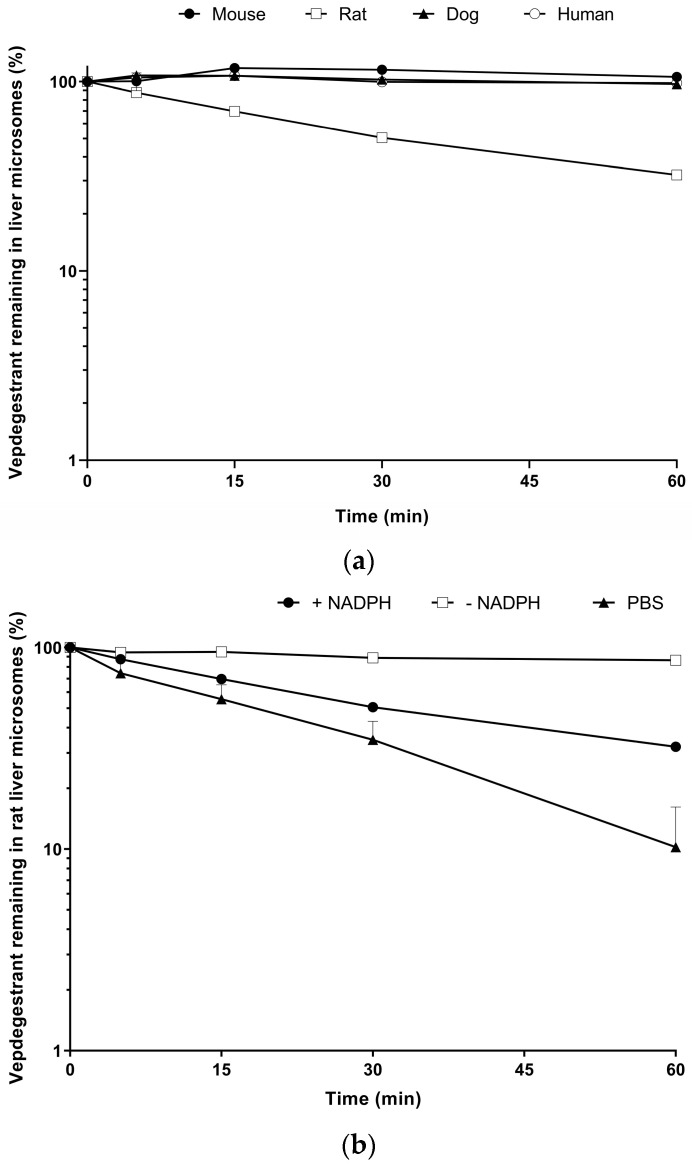
The remaining amount of vepdegestrant (%) in mouse (●), rat (□), dog (▲), and human (○) liver microsomes (**a**) and the remaining amount of vepdegestrant (%) in rat liver microsomes with NADPH (●), without NADPH (□), and in PBS (▲) (**b**). Data represent the mean ± standard deviation (*n* = 3).

The stability profiles of vepdegestrant in mouse, rat, dog, and human microsomes are shown in Figure 6a. Following 60 min incubation of vepdegestrant (1 μM) with mouse, rat, dog, and human microsomes, the remaining percentages of unconverted vepdegestrant were 106%, 32.2%, 97.1%, and 98.3%, respectively. The half-lives of vepdegestrant were 36.9, 591, and 802 min in rat, dog, and human microsomes, respectively, exhibiting high stability in all species except rats and moderate stability in rats. In mouse microsomes, the elimination half-life of vepdegestrant could not be determined, because vepdegestrant was not eliminated. Because the microsomal stability test used PBS, it was predicted that vepdegestrant would have low stability in the microsomes. However, it showed moderate to high stability in all species, likely attributed to strong non-specific binding to liver microsomes, as shown in Figure 6b. Specifically, after 1 h of incubation in PBS, only 10.2% of vepdegestrant remained. In contrast, in the absence of NADPH, 86.6% of the vepdegestrant remained in rat microsomes after 1 h of incubation. These results suggest that vepdegestrant binds to microsomal proteins, with some unbound forms potentially adsorbed to the test tube walls. Therefore, the rat microsomal stability results indicate a combination of degradation by microsomes and adsorption. However, in species other than rats, vepdegestrant levels did not decrease after 1 h of incubation, indicating very strong binding between vepdegestrant and mouse, dog, and human microsomes. Therefore, it is necessary to investigate the unbound fraction of each species.

### 2.4. Rodent Pharmacokinetic Studies

**Figure 7:**
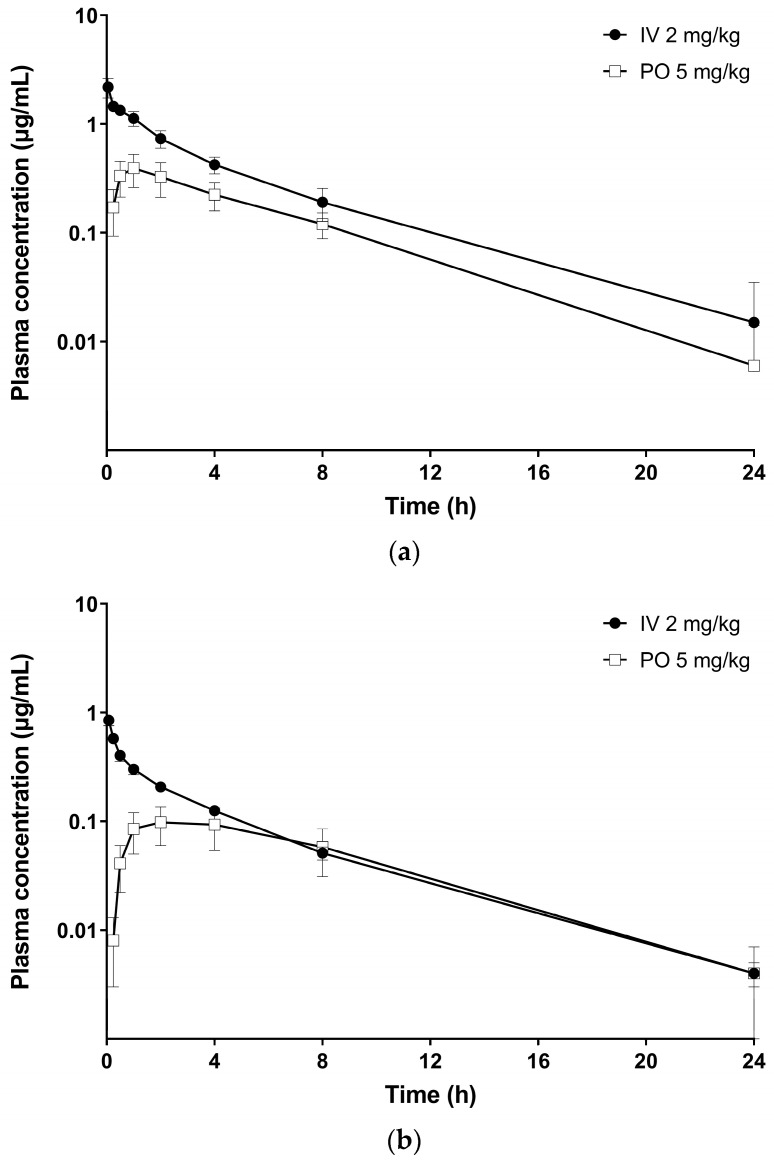
Plasma concentration–time profiles of vepdegestrant following intravenous administration at 2 mg/kg (●) and oral administration at 5 mg/kg (□) to male ICR mice (**a**) and SD rats (**b**). Data represent the mean ± standard deviation (*n* = 6 for mice and 5 for rats).

The concentration–time profiles of vepdegestrant after intravenous injection at 2 mg/kg or oral administration at 5 mg/kg in mice and rats are shown in Figure 7 and the PK parameters are summarized in Table 4.

In mice, following an intravenous injection at a dose of 2 mg/kg, clearance (CL) was 313.3 ± 44.2 mL/h/kg and the volume of distribution (V_ss_) was 1434 ± 472 mL/kg. The area under the curve from 0 to infinity (AUC_inf_) was 6.507 ± 1.057 μg/h/mL, and the half-life (T_1/2_), respectively. After oral administration of vepdegestrant at a dose of 5 mg/kg, AUC_inf_ was 2.913 ± 0.707 μg/h/mL, T_1/2_ was 3.637 ± 1.399 h. The oral bioavailability in mice was calculated to be 17.91 ± 4.35%. In rats, following intravenous injection at a dose of 2 mg/kg, CL was 1053 ± 49 mL/h/kg and V_ss_ was 4432 ± 300 mL/kg. In addition, AUC_inf_ was 1.902 ± 0.090 μg/h/mL, and T_1/2_ was 3.970 ± 0.284 h. After the oral administration of vepdegestrant at a dose of 5 mg/kg, AUC_inf_ was 1.147 ± 0.446 μg/h/mL, and T_1/2_ was 4.068 ± 0.418 h. The oral bioavailability in rats was 24.12 ± 9.39%.

The clearance in mice was low at 5.8% of liver blood flow (5.4 L/h/kg, [26]), whereas in rats, the clearance was moderate at approximately 31.9% of liver blood flow (3.3 L/h/kg, [26]). These results indicated that vepdegestrant showed high metabolic stability in mice and moderate metabolic stability in rats, which correlated well with the results of the metabolic stability tests. The volume of distribution in mice and rats was greater than 1 L/kg, suggesting good tissue distribution [27]. Vepdegestrant was expected to have relatively higher bioavailability (>30%) than other PROTAC compounds because it structurally contains the lenalidomide-derived cereblon E3 ligand [28]. However, it exhibited low to moderate oral bioavailability in rodents. These results were likely attributable to low absorption in the gastrointestinal (GI) tract, and intestinal first-pass metabolism rather than the liver first-pass effect, considering the low-to-moderate clearance values compared with the hepatic blood flow rate. Incomplete absorption could be attributed to the low solubility and permeability of vepdegestrant owing to its high molecular weight. Another possibility is the intestinal first-pass effect, which involves the degradation by intestinal microbes or enzymes, necessitating further research. Furthermore, vepdegestrant exhibited higher oral bioavailability in rats than in mice. However, considering previous studies indicating that rats exhibited lower PROTAC absorption than mice [29] and higher microsomal stability in mice than in rats, the higher oral bioavailability observed in rats could be attributed to interspecies differences. Specifically, vepdegestrant may exhibit reduced stability within the gastrointestinal tract of mice or undergo more extensive metabolism by intestinal enzymes before reaching systemic circulation following oral administration. However, further studies are required to elucidate these underlying mechanisms.

## 3. Materials and Methods

### 3.1. Materials

Vepdegestrant (ARV-471; C_45_H_49_N_5_O_4_; MW = 723.918; purity = 97.15%) was purchased from Target Mol (Wellesley Hills, MA, USA). Bavdegalutamide (ARV-110; IS; C_41_H_43_ClFN_9_O_6_; MW = 812.29) was provided by Ubix Therapeutics Co. Ltd. (Seoul, Republic of Korea). HPLC-grade acetonitrile (cat. no. UN1648) and methanol (cat. no. UN1230) were purchased from J.T. Baker (Phillipsburg, NJ, USA). HPLC grade water (cat. no. W0054) was obtained from Samchun Chemicals (Pyeongtaek, Republic of Korea). Blank heparinized plasma was prepared from male ICR mice and Sprague–Dawley (SD) rats in our laboratory. Male CD-1 mice (cat. no. 452701), male SD rats (cat. no. 452501), male beagle dogs (cat. no. 452601), and a mixed pooled human (cat. no. 452161) liver microsomes were purchased from Gentest^®^ (Woburn, MA, USA). The NADPH regeneration system (cat. no. V9510; Promega, Madison, WI, USA), ammonium formate (cat. no. 25030S0401; Junsei Chemical, Tokyo, Japan), dimethyl sulfoxide (DMSO; cat. no. D2660; Sigma-Aldrich, St. Louis, MO, USA), cremophor EL (cat. no. C5135; Sigma–Aldrich, St. Louis, MO, USA), polyethylene glycol 400 (PEG 400; cat. no. P0638; Samchun Chemicals, Pyeongtaek, Republic of Korea), and 1× PBS (cat. no. 10-010-023; Thermo Fisher Scientific, Waltham, MA, USA) were used in this study. 

### 3.2. Selection of Internal Standard

The IS corrects for variability within the sample, compensates for changes in the sensitivity of the analytical instrument, and assists in the quantification of the vepdegestrant. Bavdegalutamide was selected as the IS in this study because it has similar physicochemical properties to vepdegestrant and could be separated from vepdegestrant and detected in the analytical instrument. The IS was added at a consistent concentration to all biological samples, calibration standards, and quality control samples. The area ratio, obtained by dividing the peak area of the vepdegestrant by that of the IS, was used to construct the calibration curve, which was subsequently used to determine the concentration of vepdegestrant in the biological samples.

### 3.3. Analytical Methods

To quantify vepdegestrant in biological samples, an Agilent 1100 HPLC series (Agilent Technologies, Santa Clara, CA, USA) coupled with an API 4000 QTrap (AB SCIEX, Framingham, MA, USA) was used via an ESI interface in the positive ionization mode. Ion detection was performed in multiple reaction monitoring (MRM) mode with mass-to-charge ratios (*m*/*z*) of 724.3 → 396.3 for vepdegestrant and 813.4 → 452.2 for the IS. The column used was a Zorbax Eclipse XDB-C18 (3.5 μm, 2.1 × 150 mm; Agilent, Santa Clara, CA, USA), maintained at 40 °C. The mobile phase consisted of a mixture of 10 mM ammonium formate in distilled water and ACN (30:70, *v*/*v*) at a flow rate of 0.3 mL/min, with a sample injection volume of 5 µL. The autosampler temperature was set to 10 °C. The tandem mass spectrometry system parameters were set as follows: Ion spray voltage of 5500 V, source temperature of 600 °C, nebulizer gas pressure of 50 psi, turbo gas of 50 psi, entrance potential of 10 V, declustering potential of 76 and 121 V, and collision energies of 53 and 59 V for vepdegestrant and IS, respectively. Peak areas were automatically integrated using the Analyst software version 1.6.4 (Applied Biosystems/MDS SCIEX, Framingham, MA, USA).

### 3.4. Preparation of the Standard and Quality Control Samples

The vepdegestrant was stored at −20 °C. A stock solution (1 mg/mL) was prepared in methanol. Calibration standard and quality control (QC) samples were prepared using serial dilution with ACN to obtain 10-fold concentrated working standard solutions (10, 20, 50, 100, 1000, 2000, 5000, and 10,000 ng/mL) and QC solutions (30, 300, and 9000 ng/mL). Protein precipitation was used as the pretreatment method. Mouse or rat blank plasma samples (18 µL) were spiked with a 10-fold concentrated working solution (2 µL) and mixed. Subsequently, IS (20 µL, 300 ng/mL in ACN) and ACN (160 µL) were added. The mixture was vortexed vigorously for 10 min and centrifuged at 17,054 g under room temperature (25 °C) for 10 min. Subsequently, the supernatant (100 µL) was taken, and 5 µL was injected for HPLC-MS/MS analysis. 

### 3.5. Method Validation

Quantitative analysis of vepdegestrant in mouse and rat plasma was fully validated according to the FDA and EMA guidelines. 

Selectivity and specificity were evaluated by analyzing blank mouse and rat plasma from at least six sources to ensure no endogenous substances were present in the analyte. This involved analyzing and comparing blank and spiked samples and incurred samples obtained from rodents after vepdegestrant administration. Per the guidelines, no interference at the analyte and IS retention times in both the spiked and incurred samples should be ensured. 

The LLOQ is defined as the lowest concentration on the calibration curve where the analyte can be reliably measured. At the LLOQ, the analyte response should be at least five times higher than that of the blank signal.

Linearity was assessed using a calibration curve composed of eight concentrations (1, 2, 5, 10, 100, 200, 500, and 1000 ng/mL). Least squares linear regression with a weighting factor (1/x^2^) was used to fit the calibration curve. The correlation coefficient (r^2^) was used to confirm linearity, with an acceptance criterion of r ≥ 0.990.

Carryover was evaluated by injecting a blank sample immediately after the upper limit of quantification (ULOQ) sample to ensure that analytes from previous samples did not appear in subsequent analyses. The peak area of vepdegestrant in the blank sample should be less than 20% of the LLOQ.

Precision and accuracy were evaluated at LLOQ (1 ng/mL), LQC (3 ng/mL), MQC (30 ng/mL), and HQC (900 ng/mL). The intra-day precision and accuracy were assessed by analyzing five replicates within a single day, whereas the inter-day precision and accuracy were assessed by analyzing five replicates per day over three consecutive days. Precision and accuracy were estimated using the %CV and %RE, respectively, between the calculated and nominal concentrations. Guidance acceptance criteria were within ±15%, except for the LLOQ, which was within ± 20%.

Stability was tested for vepdegestrant in biological samples at LQC and HQC under specific conditions: Short-term stability at room temperature for 6 h, three freeze–thaw cycles, long-term stability at −20 °C for 4 weeks, and pretreated samples in an autosampler for 24 h. The stability test was performed via five replicates analysis, with the acceptance criteria being an RE within ±15% at LQC and HQC levels.

The matrix effect is the direct or indirect influence of substances present in biological samples on the response of the analyte or IS. Recovery was assessed with the analyte retrieved from the biological sample through the pretreatment steps. Although 100% recovery was not required, the recovery should be consistent and reproducible. The process efficiency was assessed to evaluate the overall efficiency of the pretreatment steps. This parameter reflected the combined effects of recovery and matrix effect. The matrix effect, recovery, and process efficiency were evaluated at the three QC levels, as previously described [30]. In this study, vepdegestrant dissolved in ACN represented set 1, vepdegestrant added to the supernatant after pre-treatment of blank plasma represented set 2, and the pre-treated vepdegestrant sample presented set 3. The matrix effect was evaluated by comparing sets 1 and 2, the recovery was assessed by comparing sets 2 and 3, and the process efficiency was evaluated by comparing sets 1 and 3.

Dilution integrity means diluting a sample with the same matrix does not affect the analysis if the concentration exceeds the ULOQ. Mouse and rat plasma were spiked with vepdegestrant to prepare test samples at final concentrations of 9000 and 27,000 ng/mL, respectively. These samples were then diluted 10- and 30-fold and compared with the HQC samples. Dilution integrity was evaluated through five replicate analyses, with acceptance criteria set for a CV of ≤15% and an RE of ≤15%.

### 3.6. In Vitro Stability Tests

The physicochemical stability of vepdegestrant was determined using various pH buffers (pH 2.0–10.0) and plasma and microsomes. 

A solution of vepdegestrant in methanol (10 µL) was added to buffer solutions at pH 2, 4, 6, 8, and 10 or pH 7.4 PBS (490 µL), yielding a final concentration of 0.1 µg/mL, and incubated at 37 °C, with 100 rpm in a water bath. At 0, 0.5, 1, and 2 h post-incubation, the incubation mixture (50 µL) was transferred to microtubes containing ice-cold ACN solution (200 µL) with IS to terminate the reaction. The mixture was vortexed thoroughly for 10 min. Subsequently, the supernatant (100 µL) was taken, and 5 µL was injected for HPLC-MS/MS analysis.

Plasma stability of vepdegestrant was investigated in mouse and rat plasma. Vepdegestrant (10 µL) was added to blank mouse or rat plasma (490 µL) to prepare a final concentration of 0.1 µg/mL. The mixtures were incubated at 4, 25, and 37 °C. After 0, 1, and 4 h of incubation, aliquots (50 µL) were added to microtubes containing ice-cold ACN solution with an IS (200 µL) to terminate the reaction. The mixture was vortexed thoroughly for 10 min and centrifuged at 17,054 g under 25 °C for 10 min. Subsequently, the supernatant (100 µL) was taken, and 5 µL was injected for HPLC-MS/MS analysis.

The metabolic stability of vepdegestrant was evaluated using liver microsomes obtained from mice, rats, dogs, and humans. Vepdegestrant was dissolved in DMSO to prepare a stock solution with a concentration of 0.1 mM. Microsomes (25 µL) were spiked into PBS (440 µL) and pre-incubated for 5 min. After pre-incubation, vepdegestrant stock solution (5 µL) was added, followed by an NADPH regenerating system (27 µL) to initiate the reaction. The composition of the reaction mixture was as follows: Vepdegestrant (final concentration 1 µM), mouse, rat, dog, human liver microsomes (protein concentration: 1 mg/mL), NADPH regenerating system (final concentrations: 1.3 mM NADP^+^, 3.3 mM glucose-6-phosphate, 0.4 U/mL glucose-6-phosphate dehydrogenase, and 3.3 mM magnesium chloride), and PBS (pH 7.4). At 5, 15, 30, and 60 min after starting the reaction, aliquots (50 µL) were taken and added to ice-cold ACN with an IS (200 µL) to terminate the reaction. For the zero-time samples, aliquots were taken before adding NADPH. Subsequently, the mixture was vigorously mixed for 10 min and centrifuged at 17,054 *g* under 25 °C for 10 min. For analysis, 5 µL of the supernatant was injected into the HPLC-MS/MS system. In addition, the stability of vepdegestrant in rat liver microsomes was assessed in the absence of NADPH or NADPH and microsomes (PBS only). The experimental procedure was the same as described for the microsomal stability test, except that an equivalent volume of PBS was added instead of NADPH or microsomes.

### 3.7. Rodent Pharmacokinetic Studies

Twelve healthy male ICR mice (6 weeks old, 27.5–30.5 g) and 10 healthy male Sprague–Dawley (SD) rats (7 weeks old, 195.5–212.0 g) were acquired from Orient Bio Inc. (Seongnam, Republic of Korea) and used for PK studies. The animals were kept in rooms in a temperature range of 20–25 °C, humidity of 40–60%, and a 12-h light/dark cycle. Before the experiment, mice were fasted for 4 h, whereas rats were fasted for 14 h, with only water provided during the fasting period. After 4 h of drug administration, animals were provided with free access to food and water. All animal experiments were approved by the Institutional Animal Care and Use Committee of the Chungnam National University (202310A-CNU-178; Daejeon, Republic of Korea).

For the PK studies in mice, a vepdegestrant dosing solution was prepared using a combination of DMSO, PEG400, and saline (5:25:70, *v*/*v*/*v*). The dosing solution was administered intravenously (IV, 2 mg/kg) through the tail vein or orally (PO, 5 mg/kg) through a gavage needle. The dose volume for both routes was 10 mL/kg. Blood samples (50 μL) were collected via the retro-orbital plexus at 0.05 (IV only), 0.25, 0.5, 1, 2, 4, 8, and 24 h post vepdegestrant administration using heparin-coated microcapillaries. Blood samples were immediately centrifuged at 12,032 g under 25 °C for 5 min, and plasma aliquots (20 μL) were stored at −20 °C until HPLC-MS/MS analysis.

For the PK studies in rats, a vepdegestrant dosing solution was prepared using a combination of DMSO, PEG 400, cremophor EL, and saline (5:40:2.5:52.5, *v*/*v*/*v*/*v*). The dosages were set at 2 and 5 mg/kg for IV and PO, respectively, consistent with mouse PK studies. The dose volumes were 2 and 5 mL/kg IV and PO, respectively. Blood samples (100 µL) were collected from the jugular vein using heparinized syringes at 0.08 (IV only), 0.25, 0.5, 1, 2, 4, 8, and 24 h post vepdegestrant administration. Blood samples were immediately centrifuged at 12,032 g under 25 °C for 5 min, and plasma aliquots (20 μL) were stored at −20 °C until HPLC-MS/MS analysis.

The collected plasma samples from mice or rats (20 µL) were added with IS (20 µL) and ACN (160 µL). Subsequently, the mixture was vigorously mixed for 10 min and centrifuged at 17,054× *g* under 25 °C for 10 min. An aliquot of the supernatant (100 μL) was taken, and a volume of 5 μL was injected for HPLC-MS/MS analysis. PK parameters were calculated using Phoenix^®^ 8.3 software (Certara L.P., Princeton, NJ, USA), as described previously [20,30]. 

## 4. Conclusions

In conclusion, a precise and sensitive HPLC-MS/MS method was developed and validated for the detection and quantification of vepdegestrant in mouse and rat plasma. The reported analytical method met all EMA and FDA requirements, ensuring reliability and reproducibility. This validated method was successfully applied in PK studies in rodents, revealing that vepdegestrant remained stable in mouse and rat plasma, regardless of temperature. In liver microsomes, vepdegestrant exhibited high stability in samples from mice, beagle dogs, and humans and moderate stability in those from rats. However, vepdegestrant was extensively unstable under conditions ranging from pH 4 to 10 and moderately unstable under pH 2. This finding suggests that vepdegestrant may adsorb onto microtubes when buffer solutions are used instead of organic solvents. In PK studies in mice and rats, vepdegestrant showed low-to-moderate clearance and a moderate volume of distribution, with low-to-moderate bioavailability owing to limited absorption. Despite its low bioavailability, vepdegestrant has progressed to clinical development owing to its stability in plasma, status as a relatively large-molecule PROTAC, and anticancer properties. Overall, these findings aid in understanding the PK properties of vepdegestrant and could serve as a reference for future PROTAC drug development.

## Figures and Tables

**Table 1:** Intra- and inter-day precisions and accuracies for vepdegestrant in mouse and rat plasma.

Nominal Concentration (ng/mL)	Measured Concentration (ng/mL)	Precision (CV, %)	Accuracy (RE, %)
Mouse plasma			
Intra-day (*n* = 5)			
1	1.029 ± 0.105	8.33	2.86
3	3.338 ± 0.355	10.64	11.27
30	33.60 ± 3.21	9.54	12.00
900	1007 ± 78	7.70	11.91
Inter-day (*n* = 15)			
1	1.057 ± 0.142	13.43	5.69
3	3.271 ± 0.210	6.43	9.04
30	31.47 ± 3.83	12.16	4.91
900	898.1 ± 86.4	9.62	0.21
Rat plasma			
Intra-day (*n* = 5)			
1	1.021 ± 0.109	10.63	2.10
3	2.974 ± 0.218	7.32	0.87
30	30.10 ± 2.78	9.30	0.33
900	905.6 ± 48.4	5.35	0.62
Inter-day (*n* = 15)			
1	1.023 ± 0.132	12.94	2.27
3	3.033 ± 0.365	12.02	1.11
30	31.15 ± 4.19	13.43	3.84
900	925.9 ± 109.9	11.87	2.87

CV, coefficient of variation; RE, relative error.

**Table 2:** Stability of vepdegestrant in mouse and rat plasma.

Storage Conditions	Nominal Concentration (ng/mL)	Stability in Mouse Plasma (%)	Stability in Rat Plasma (%)
6 h at room temperature (25 °C)	3	97.70 ± 4.12	100.3 ± 7.1
	900	110.7 ± 2.8	99.59 ± 4.12
Freeze–thaw three-cycle (−20 °C → RT)	3	99.78 ± 1.60	98.70 ± 7.09
	900	103.3 ± 4.1	94.98 ± 1.40
1 month at −20 °C	3	103.1 ± 5.4	98.83 ± 12.58
	900	107.7 ± 4.0	104.3 ± 8.5
Processed sample in 10 °C autosampler for 24 h	3	104.0 ± 7.2	99.86 ± 0.84
	900	98.49 ± 8.79	92.18 ± 6.32

Data presented as mean ± standard deviation (*n* = 5).

**Table 3:** Matrix effect, recovery, and process efficiency of vepdegestrant in mouse and rat plasma.

Nominal Concentration (ng/mL)	Matrix Effect (%)	Recovery (%)	Process Efficiency (%)
Mouse plasma			
3	106.8 ± 4.86	92.89 ± 2.12	99.16 ± 2.26
30	108.8 ± 2.13	95.95 ± 0.49	104.4 ± 0.53
900	92.02 ± 4.76	106.0 ± 3.91	97.58 ± 3.60
IS (300 ng/mL)	92.66 ± 7.72	94.73 ± 4.94	87.78 ± 4.57
Rat plasma			
3	91.35 ± 8.96	93.34 ± 5.31	88.64 ± 5.05
30	90.78 ± 6.60	88.54 ± 3.09	81.45 ± 2.84
900	92.26 ± 3.00	85.94 ± 2.61	87.80 ± 2.66
IS (300 ng/mL)	91.99 ± 6.23	90.05 ± 7.45	87.60 ± 7.25

Data presented as mean ± standard deviation (*n* = 5).

**Table 4:** Pharmacokinetic parameters of vepdegestrant following intravenous and oral administration in fasted male ICR mice and SD rats.

Parameters	Mouse		Rat	
	IV	PO	IV	PO
T_max_ (h)	0.050 ± 0.000	1.000 ± 0.000	0.083 ± 0.000	3.200 ± 2.950
C_max_ (μg/mL)	2.180 ± 0.442	0.393 ± 0.133	0.850 ± 0.095	0.108 ± 0.038
T_1/2_ (h)	3.790 ± 1.151	3.637 ± 1.399	3.970 ± 0.284	4.068 ± 0.418
AUC_last_ (μg/h/mL)	6.396 ± 1.080	2.869 ± 0.704	1.881 ± 0.084	1.123 ± 0.428
AUC_inf_ (μg/h/mL)	6.507 ± 1.057	2.913 ± 0.707	1.902 ± 0.090	1.147 ± 0.446
CL (mL/h/kg)	313.3 ± 44.2	-	1053 ± 49	-
V_ss_ (mL/kg)	1434 ± 472	-	4354 ± 300	-
MRT (h)	4.607 ± 1.431	5.581 ± 1.344	4.140 ± 0.348	6.541 ± 0.965
BA (%)	-	17.91 ± 4.35	-	24.12 ± 9.39

IV, intravenous; PO, per oral; C_max_, maximum plasma concentration; T_max_, time to reach C_max_; T_1/2_, terminal elimination half-life; AUC, area under the plasma concentration–time curve; CL, systemic clearance; V_ss_, steady-state volume of distribution; MRT, mean residence time; BA, bioavailability; Data are presented as mean ± standard deviation (*n* = 6 for mice and *n* = 5 for rats).

## Data Availability

The Data presented in this study are available.
